# Cost Utility Modeling of Reducing Waiting Times for Elective Surgical Interventions: Case Study of Egyptian Initiative

**DOI:** 10.3390/healthcare13131619

**Published:** 2025-07-07

**Authors:** Ahmad Nader Fasseeh, Amany Ahmed Salem, Ahmed Yehia Khalifa, Asmaa Khairy ElBerri, Nada Abaza, Baher Elezbawy, Naeema Al Qasseer, Balázs Nagy, Zoltán Kaló, Bertalan Németh, Rok Hren

**Affiliations:** 1 Syreon Middle East, Alexandria 5424041, Egypt; ahmad.fasseeh@syreon.eu (A.N.F.); nada.abaza@syreon.eu (N.A.); baher.elezbawy@syreon.eu (B.E.); 2 Faculty of Pharmacy, Alexandria University, Alexandria 5424041, Egypt; 3 Manchester Centre for Health Economics, The University of Manchester, Manchester M13 9PL, UK; 4 Faculty of Medicine, Cairo University, Cairo 11562, Egypt; 5 World Health Organization Representative Office, Cairo 11516, Egyptnaeema@alqasseer.com (N.A.Q.); 6 Ministry of Health and Population, Cairo 11562, Egypt; asmaaelberri85@gmail.com; 7 Syreon Research Institute, 1142 Budapest, Hungary; balazs.nagy@syreon.eu (B.N.);; 8 Center for Health technology Assessment, Semmelweis University, 1085 Budapest, Hungary; 9 Faculty of Mathematics and Physics, University of Ljubljana, 1000 Ljubljana, Slovenia; 10 Institute of Mathematics, Physics, and Mechanics, Ljubljana, 1000 Ljubljana, Slovenia

**Keywords:** waiting times, cost-effectiveness, elective surgery, health system reform, economic evaluation

## Abstract

**Background/Objectives:** Reducing waiting times for elective surgeries remains a critical global healthcare challenge that negatively impacts patient outcomes and economic productivity. This study develops an adaptable cost-utility modeling framework for assessing the cost-effectiveness (CE) of reducing waiting time for elective surgeries in data-limited environments. **Methods:** We evaluated the economic and health impacts of Egypt’s recent initiative aimed at decreasing surgical waiting lists. The study conducts a CE analysis of the initiative by estimating incremental costs (expressed in Egyptian Pounds—EGP) and outcomes (expressed in quality-adjusted life years—QALYs) before and after its implementation, performs a benefit–cost analysis to quantify the initiative’s return on investment, and employs a budget share method to evaluate catastrophic health expenditure (CHE). The analysis included five elective surgical interventions: open-heart surgery, cardiac catheterization, cochlear implantation, ophthalmic surgery, and orthopedic (joint replacement) surgery. **Results:** The main research outcomes of the study are as follows. The initiative resulted in incremental cost-effectiveness ratios of EGP 46,795 (societal perspective) and EGP 56,094 (payer perspective) per QALY, both within acceptable CE thresholds. Most of the evaluated interventions demonstrated substantial returns on the investment. Without public funding, more than 90% of patients faced CHE, indicating considerable financial barriers to elective surgeries. **Conclusions:** Egypt’s initiative to reduce waiting times was deemed cost-effective. Our adaptable modeling framework could be practical for similar evaluations in low/middle-income countries, especially where data is limited. Scaling up the initiative to include additional curative and preventive services and integrating it with broader health system reforms in Egypt is strongly recommended.

## 1. Introduction

Waiting lists for elective surgeries and procedures represent a critical global healthcare challenge [1,2]. Prolonged waiting times persist across healthcare systems worldwide [1,3], adversely affecting patient well-being and economic productivity [2]. Delays in essential surgical interventions—such as cardiac surgery, cochlear implantation, joint replacement, and ophthalmic procedures—frequently exceed clinically acceptable thresholds, leading to poor health outcomes [4]. These delays can cause irreversible physical harm, significantly reduce quality of life, lead to permanent disability, and in severe cases, result in death [5,6,7]. For example, postponed cardiac procedures increase the risk of complications, hospital admissions, and mortality [8]. Similarly, delays in cochlear implantation among children impede auditory and cognitive development, with long-term consequences for social integration and educational achievement [9].

Extended waiting times have considerable economic consequences, including increased direct medical costs and indirect productivity losses [1,10]. Patients awaiting surgery frequently suffer from physical limitations that restrict their capacity to work, imposing financial stress on themselves and their families. Caregivers also often experience economic hardship due to reduced working hours or having to exit the workforce entirely to provide support [11]. This dual burden—on patients and caregivers—results in a broader economic impact, contributing to diminished household income and, ultimately, reduced national productivity.

A recent Organization for Economic Cooperation and Development (OECD) report emphasized significant disparities in waiting times for three common elective surgeries—cataract surgery, hip replacement, and knee replacement—across twelve jurisdictions [12]. For cataract surgery, median waiting times exceeded 100 days in Ireland and surpassed 200 days in Costa Rica and Slovenia, whereas Hungary, Spain, and Sweden reported median waits below 50 days. Similar patterns were observed for hip and knee replacements, with median waiting times exceeding 400 days in Chile and over 700 days in Poland. Specifically, median waiting times for knee replacements were over 600 days in Slovenia and Costa Rica and reached 900 days in Poland. Only Spain maintained a median wait times under 100 days. These findings point to the global scope of the surgical waiting list challenges, which typically arise from both the demand and supply constraints within healthcare systems [3,13]. In response, many countries facing prolonged waiting times have implemented a variety of policy measures to mitigate their adverse effects [14,15,16].

Egypt’s healthcare system faces similar challenges, including extended wait times for elective procedures. According to the Ministry of Health and Population (MoHP), waiting periods for certain surgeries frequently exceed one year, causing deteriorating health outcomes, income loss, and increased financial burdens on households. In response, the MoHP launched a national initiative in July 2018 (hereafter referred to as the “MoHP initiative”) aimed at eliminating or significantly reducing waiting lists for high-priority surgical procedures. The MoHP initiative included expanding service provider networks and allocating additional resources to expedite critical surgeries.

Interventions designed to reduce surgical waiting times generally require increased healthcare resources, including trained personnel, infrastructure, and sustained funding [17]. Priority should be given to surgeries where delays pose the greatest risk to health and economic outcomes. Conducting cost-effectiveness analyses of these interventions can provide valuable evidence for healthcare decision-makers to allocate these resources efficiently.

Evaluating such interventions is inherently complex, requiring measurement of both costs and health outcomes before and after implementation. This challenge is further amplified in data-scarce settings, where the lack of reliable information hinders systematic evaluation. In such settings, the availability of a dedicated framework and set of guidelines for evaluating the benefits of reducing waiting times for elective surgeries could significantly simplify the assessment process and assist healthcare decision-makers in implementing cost-effective interventions.

This study develops an adaptable framework for assessing the cost-effectiveness of reducing waiting time for elective surgeries, especially designed for data-limited environments. Specifically, the proposed model evaluates the impact of Egypt’s national MoHP initiative aimed at shortening surgical waiting lists, providing a practical and transferable tool for other countries facing similar challenges.

## 2. Materials and Methods

### 2.1. Model Framework

The conceptual foundation of our model was established through a systematic literature review of economic evaluations targeting reductions in surgical waiting times [18]. The model was deliberately designed to be comprehensive, adaptable, and generalizable, allowing its application across diverse surgical procedures and healthcare settings, particularly those facing limited data availability. It integrates cost-effectiveness and cost–benefit methodologies to assess both the health and economic outcomes of initiatives aimed at reducing waiting times [18]. Importantly, our model emphasizes the incremental difference between early and delayed surgical interventions, rather than absolute values for each scenario. It does not attempt to quantify the overall mortality benefit or total surgical costs but rather focuses on the value of earlier intervention compared to delayed treatment. This simplified structure substantially reduces data requirements, making the model particularly suitable for resource-constrained settings.

### 2.2. Model Structure

The model estimates incremental health outcomes and costs associated with reduced surgical waiting times by comparing two primary patient pathways: (i) early surgery, and (ii) delayed or missed surgery. Health outcomes are expressed in quality-adjusted life years (QALYs) and are evaluated over a patient’s lifetime. The patient’s timeline is segmented into intervals reflecting divergences in health status between the two arms, allowing for the clear attribution of incremental effects.

**Figure 1:**
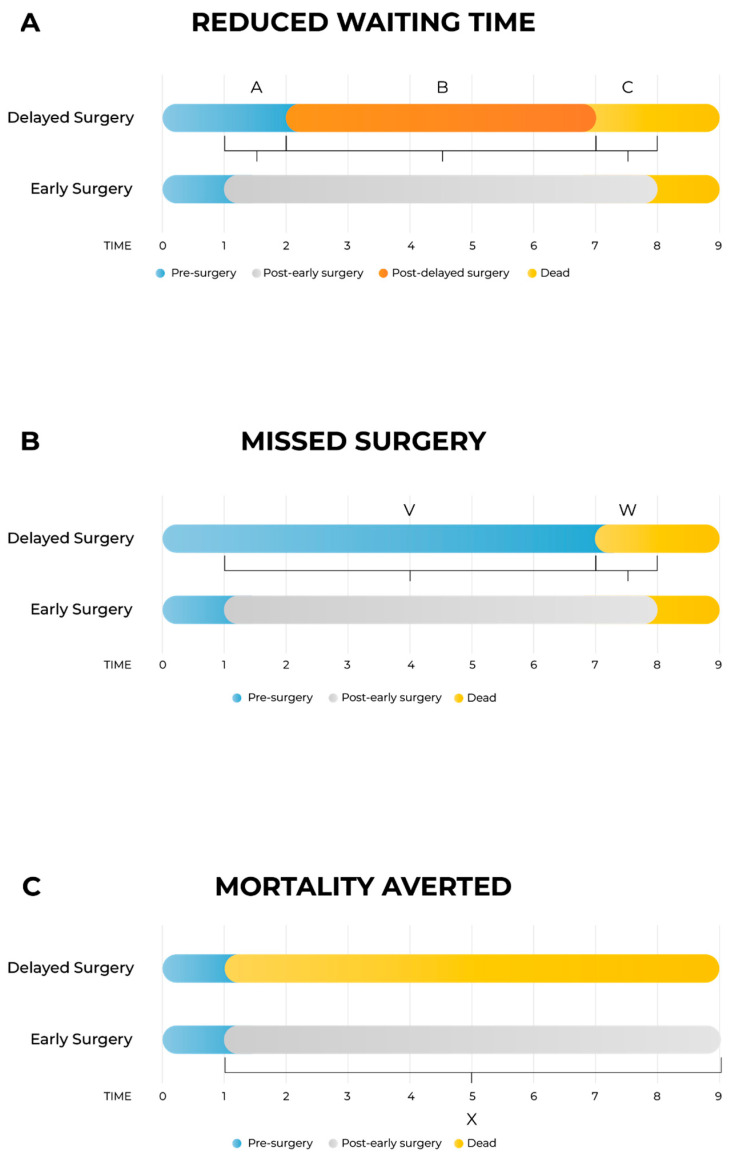
Schematic presentation of the model. Figure 1A: The model is divided into delayed surgery arm and early surgery arm. There are four health states: pre-surgery, post-delayed surgery, post-early surgery and death; three health states are on each arm as post-delayed surgery is exclusive to the delayed arm, and post-early surgery is exclusive to the early arm. (**A**) Time between early and delayed intervention; (**B**) time between delayed intervention and death; (**C**) time between death in the delayed surgery arm and death in the early surgery arm. Figure 1B: Schematic presentation of the model for the case when patients missed the surgery entirely. The segment V is time between early intervention and death in the missed surgery arm (corresponding to the segment A when the segment B is zero in Figure 1A), and the segment W is time between death in the missed surgery arm and death in the early surgery arm (corresponding to the segment C when the segment B is zero in Figure 1A). Figure 1C: Schematic presentation of the model for the special case when surgery is lifesaving. In this model, there are no segments A and B from Figure 1A or segment V from Figure 1B, and the segment X is time between death in the no-surgery arm and death in the surgery arm (corresponding to the segment C in Figure 1A).

As shown in Figure 1A, the model differentiates between three distinct health states per arm: pre-surgery, post-surgery (either early or delayed), and death. Three primary time intervals capture the incremental impacts:

- Segment A: time interval between early and delayed surgery, capturing the quality-of-life (QoL) gap while the delayed group awaits surgery;
- Segment B: post-surgery interval to death, accounting for potential QoL differences after both groups have undergone surgery;
- Segment C: survival gap interval, capturing QALYs lost due to earlier mortality in the delayed arm compared to the early surgery arm.

In cases where surgery is entirely missed (Figure 1B), the structure simplifies into two segments:

- Segment V: time from early intervention until death in the missed surgery arm;
- Segment W: time gap between deaths in missed and early surgery arms.

For life-saving surgeries (Figure 1C), only one segment (Segment X) is included, representing the time from immediate death due to no surgery until death in the early surgery arm. In this scenario, to reduce data requirements, death in the no-surgery arm is assumed to coincide with the scheduled early surgery date, an approximation deemed acceptable given its minimal impact on overall outcomes.

The model incorporates direct and indirect costs, enabling evaluation from both the payer and societal perspectives. Effectiveness in QALYs includes gains in QoL from shorter waiting times, improved post-surgical outcomes, and increased life expectancy resulting from timely intervention. Pre-operative stress, reflecting QoL decrements during prolonged waiting periods, is explicitly considered in Segment A. QALYs are thus accurately represented as areas under the health-state curves.

Patients are classified into three distinct subgroups based on their clinical outcomes:

- Reduced Waiting Time (RWT): patients undergoing surgery after shorter waits;
- Avoided Missed Surgery (AMS): patients receiving timely intervention through the MoHP initiative and who would have become otherwise ineligible for surgery due to disease progression;
- Mortality Averted (MA): patients receiving earlier access to surgery and who would have otherwise died while waiting.

Analyses explicitly include five elective surgical interventions targeted by the MoHP initiative: open-heart surgery, cardiac catheterization, cochlear implantation, ophthalmologic (retinal) surgery, and orthopedic (joint replacement) surgery. No emergency procedures were considered. All mathematical equations and parameter specifications used in the model are provided in Appendix A.

### 2.3. Model Parameters

**Figure A1:**
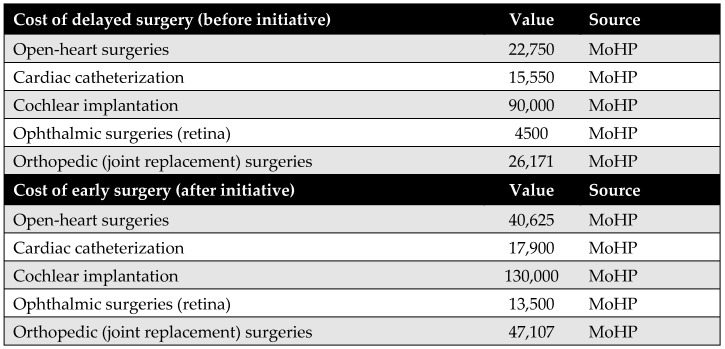
Surgical costs before and after the MoHP initiative.

Comprehensive descriptions of the model parameters are provided in the appendices: cost parameters in Appendix B, utility parameters in Appendix C, population parameters in Appendix D, and productivity parameters in Appendix E. Due to the lack of reliable local data, utility values were derived from internationally published literature, in line with standard practice. Notably, surgical costs before and after the MoHP initiative were obtained directly from the MoHP, ensuring a high level of reliability, as these figures are based on actual payments rather than estimates. Detailed cost data are presented in Figure A1 of Appendix B. 

### 2.4. Time Horizon and Discounting

The model adopts a lifetime time horizon, beginning with the perioperative period. In accordance with national guidelines [19], both costs and QALYs are discounted at an annual rate of 3.5%.

### 2.5. Model Validation

Although external validation was not feasible due to the absence of comparable real-world data, the model underwent critical review by external health economists and clinicians. Furthermore, computational validation was performed by an independent modeler not involved in the model’s development.

### 2.6. Sensitivity Analysis

To test the robustness of the model, both a deterministic sensitivity analysis (DSA) and probabilistic sensitivity analysis (PSA) were performed using the incremental cost-effectiveness ratio (ICER) from a societal perspective, including indirect costs. The analyses were conducted for all surgeries combined, with results weighted by the number of patients in each surgical group. Total QALYs gained and total costs were aggregated across all surgeries at the population level (rather than per patient). The incremental cost was then divided by the incremental QALYs to generate a single, composite ICER representing the entire study population.

A one-way DSA was performed by varying base-case parameter estimates—such as cost inputs—by ±10%, and the most influential variables were identified using a tornado diagram. Variables included in the DSA are listed in Appendix F. The PSA accounted for parameter uncertainty by assigning probability distributions to all key inputs and performing 5000 Monte Carlo simulations; it generated distributions of expected costs and QALYs for both the early and delayed intervention arms. The following distribution assumptions were used: (i) beta for utilities, QALYs, and proportions, (ii) normal for demographic and time-related variables, and (iii) gamma for cost data. Further details on the PSA inputs are available in Appendix F.

### 2.7. Reported Outcomes

Results are reported both in aggregate format and separately for each of the five priority interventions, including both discounted and undiscounted cost estimates. Outcomes are presented per patient and for the entire study population. Costs are expressed in Egyptian Pounds (EGP), and health outcomes are measured in QALYs. A willingness-to-pay (WTP) threshold of EGP 56,000 per QALY—equivalent to one GDP per capita in 2020—is used to evaluate cost-effectiveness, in line with national recommendation [20]; it corresponds to the year in which the MoHP initiative was implemented and the cost calculations were performed. The WTP threshold was determined using the Syreon Cost-Effectiveness Threshold Calculator, applying the following settings: public perspective, orphan drug switch set to ‘No’, and an Incremental Relative QALY Gain (IRQG) multiplier of 1 [20]. The outcomes reported in the study are summarized in Table 1.

### 2.8. Calculating Catastrophic Health Expenditure (CHE)

Catastrophic health expenditure (CHE) is defined as health spending that exceeds a certain threshold of a household’s total expenditure. Several methods are available to estimate CHE. We employed the budget share method as the base-case approach for assessing CHE, in alignment with the recommended methodology for reporting on SDG Indicator 3.8.2 under the 2030 Sustainable Development Goals framework [21]. Although some critiques argue that this method may oversimplify the measurement of financial hardship [22], it remains the globally accepted standard for health system monitoring. To ensure robustness, we also tested two alternative methods—the normative and partial normative approaches—and observed broadly consistent results. According to the budget share method, out-of-pocket (OOP) healthcare payments exceeding 10% or 25% of a household’s total expenditure are classified as catastrophic. For this analysis, a 25% threshold was used as the base-case criterion for identifying financial hardship.

## 3. Results

**Figure 2:**
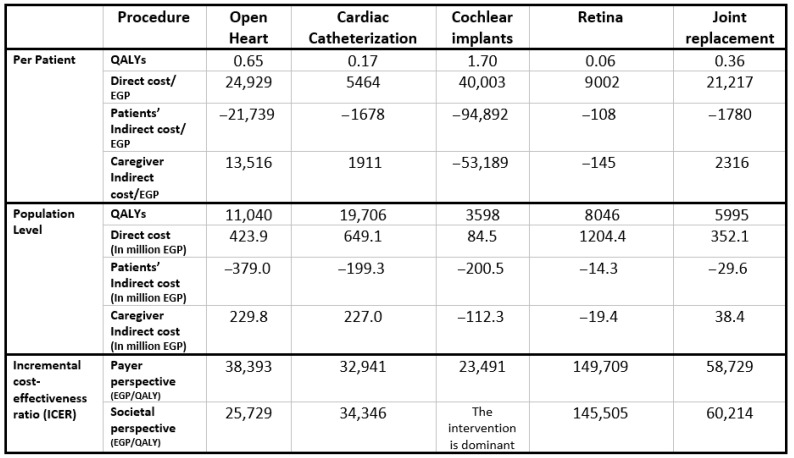
Model results for the five included surgeries.

Figure 2 presents the aggregated results across all five priority interventions at the population level, weighted by patient numbers for each surgery. The Egyptian MoHP initiative to reduce surgical waiting times resulted in a total gain of 48,385 QALYs, with an incremental cost of EGP 2,264,156,090, inclusive of productivity losses. The corresponding ICER is EGP 46,795 per QALY, which falls within Egypt’s accepted cost-effectiveness threshold. Individual intervention results and their respective benefit–cost ratios are shown in Appendix G.

**Figure 3:**
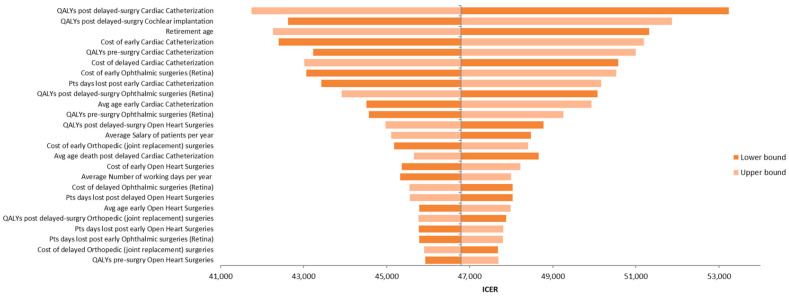
Tornado diagram illustrating the results of the deterministic sensitivity analysis (DSA).

**Figure 4:**
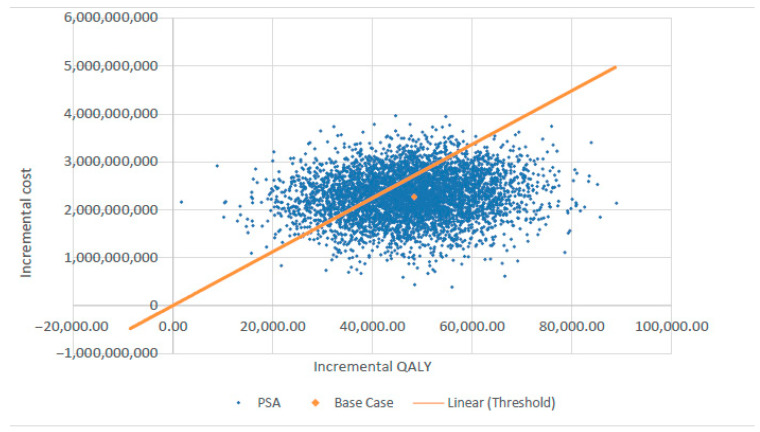
Probabilistic sensitivity analysis (PSA) scatter plot showing cost-effectiveness results. QALY—quality-adjusted life year.

Figure 3 presents the tornado diagram summarizing the DSA results from a societal perspective, indicating that the model outcomes are most sensitive to QALY estimates for delayed cardiac catheterization and cochlear implantation. The results of PSA, also from the societal perspective, are depicted in Figure 4 using a scatter plot.

**Figure 5:**
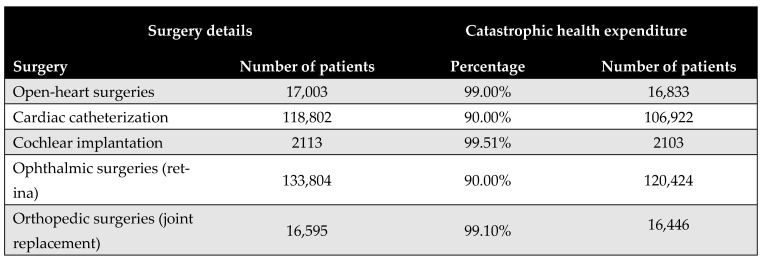
Proportion of patients experiencing catastrophic health expenditure (CHE) for the five priority interventions based on the budget share method.

Figure 5 shows the proportion of the population that would face CHE if required to pay out-of-pocket for the five priority surgical interventions. The results reveal substantial financial access barriers, with the majority of procedures deemed unaffordable for at least 90% of the eligible patient population. Cochlear implantation was identified as the least affordable, with only 0.5% of the eligible patient population able to afford it without incurring CHE. Further details are provided in Appendix H.

This study could offer a structured framework for assessing the cost-effectiveness of reducing waiting times for elective surgeries, specifically tailored for data-scarce settings. To support broader applicability, an adaptation guide has been developed, detailing model input parameters along with recommended data sources (Appendix I). The model is intentionally designed for adaptability across different countries and healthcare systems and is provided as Supplementary Materials. An illustrative application scenario is provided in Appendix J to demonstrate its potential use in diverse contexts.

## 4. Discussion

In this study, we present evidence on the economic and societal value of reducing waiting times for elective surgical procedures in Egypt. By creating a robust yet adaptable modeling framework, we were able to evaluate the cost-effectiveness and the financial protection impact of a large-scale public health initiative implemented by MoHP. Our results not only affirm the initiative’s overall cost-effectiveness from both the payer and societal perspectives but also indicate its potential to improve health equity.

A core value of this study lies in its ability to generate reliable estimates even in settings with limited data availability. The model was designed to balance analytical rigor with practical applicability, facilitating informed decision-making without requiring exhaustive micro-level data inputs. This approach is particularly valuable for low/middle-income countries (LMICs), where data constraints often hinder the application of conventional health economic evaluations. Our findings suggest that this methodology could be readily adapted to other LMIC settings facing similar challenges related to surgical wait times and constrained healthcare financing.

Our modeling study is among the few that have directly evaluated the cost-effectiveness of reducing surgical waiting times. In our accompanying systematic literature review [18], we identified only nine relevant studies across a range of elective surgical procedures [23,24,25,26,27,28,29,30,31], indicating the limited global evidence base in this area. These studies, along with our findings, consistently indicate that shorter waiting times are not only highly cost effective but often cost saving. In our analysis, all five procedures were found to be cost-effective when considered collectively, with the benefit being particularly pronounced in cochlear implantation, which showed a dominant outcome from the societal perspective and yielded the highest return on investment. Conversely, the analysis also uncovered the variability in cost-effectiveness across procedures. While open-heart surgery and cardiac catheterization were cost-effective under both evaluation perspectives, retinal surgery fell outside acceptable thresholds, likely due to its lower measurable impact on survival and productivity.

Our recent systematic review of various surgical domains revealed a consistent pattern: shorter waiting times were often associated with favorable cost-effectiveness outcomes, and in some cases, they even led to net cost savings. In musculoskeletal procedures such as total knee arthroplasty (TKA) and hip revision surgeries, earlier treatment was generally linked to lower long-term healthcare costs and enhanced patient quality of life, with the notable exception of a single study due to specific study design with a higher proportion of costly surgical procedures in the cohort of patients receiving early treatment compared to those with delayed treatment. Cardiovascular interventions, particularly transcatheter aortic valve implantation (TAVI), also benefited from reduced delays, which contributed to fewer hospital admissions and lower mortality rates among patients on waiting lists, while remaining economically justified. This trend extended to ophthalmologic and gastrointestinal operations as well—accelerated access to procedures like cataract extraction and bariatric surgery was associated with both clinical improvements and potential reductions in overall healthcare expenditure.

From the perspective of CHE, our findings reveal significant financial access barriers. The budget share analysis revealed that at least 90% of the eligible population would face CHE if required to pay out-of-pocket for any of the five priority procedures. This underlines the critical importance of publicly funded initiatives in expanding access and protecting vulnerable populations. Notably, cochlear implantation was the least affordable, with only 0.5% of households able to afford it without financial hardship—despite being the most cost-effective intervention.

These findings advocate for the integration of surgical access initiatives into broader universal health coverage (UHC) strategies. They also strengthen the case for including surgical services within essential benefit packages, especially in countries where elective procedures are often underfunded or excluded from routine public provision. Moreover, the demonstrated return on investment further justifies the expansion of such initiatives from a macroeconomic perspective.

Cochlear implantation was found to have a dominant outcome, being both more effective and cost-saving at the societal level, despite its high CHE from the household perspective. This apparent contradiction can be explained by the differing analytical viewpoints. The high CHE reflects the substantial upfront costs of the device and surgical procedure—costs that are often unaffordable for individual households without public financial support. However, when analyzed from a societal perspective, the intervention yields considerable long-term health and economic benefits, particularly due to the early age at which the procedure is typically performed. The resulting gains in QALYs, coupled with improved educational, social, and employment outcomes over a recipient’s lifetime, drive the overall cost-saving profile. This emphasizes the importance of distinguishing between short-term affordability for individuals and long-term value for society when evaluating high-cost health interventions.

Despite these positive findings, several limitations must be acknowledged. First, the societal impact of delayed or missed surgeries may be underestimated, particularly due to the lack of data on long-term productivity loss. For cochlear implants, assumptions had to be made in the absence of detailed evidence on the lifelong effects of delayed auditory rehabilitation in children. Although the disability resulting from late or missed implantation is likely to affect educational and employment outcomes, no sufficient data were available to model this impact quantitatively. Similarly, the economic burden of productivity loss related to vision impairment from delayed retinal surgeries could not be fully captured due to limited published evidence.

Additionally, the lack of localized utility data—especially regarding the health-related quality-of-life decrements associated with prolonged waiting—and the absence of detailed cost stratification by timing (early vs. delayed surgery) introduced uncertainty into some of our estimates. These data gaps limit the precision of our model.

Considering these limitations, future research should focus on improving the granularity and relevance of local input data, particularly for health utilities and productivity loss estimates, and the psychosocial impacts of delayed care. Additionally, distributional cost-effectiveness analyses (DCEAs) could be conducted to examine how benefits and costs are distributed across different socioeconomic groups, and whether initiatives like this contribute to reducing health disparities.

Finally, this study provides a foundation for the broader application of the proposed modeling framework beyond elective surgical interventions. The same methodology could be adapted to assess the economic value of reducing delays in other areas, such as diagnostic services, cancer treatment, and rehabilitation care. Expanding the use of this framework can support more efficient and equitable resource allocation, ultimately contributing to more resilient and inclusive health systems.

## 5. Conclusions

This evaluation represents a step in quantifying the economic and societal impact of large-scale health interventions such as the Egyptian public initiative of reducing wait times. The methodological approach is practical, emphasizing incremental costs and outcomes while minimizing the reliance on unavailable micro-level data. Although some assumptions were necessary due to data gaps, our findings provide strong evidence of the initiative’s cost-effectiveness. Future research will further explore the initiative’s role in enhancing healthcare access and protecting households from CHE.

## Figures and Tables

**Table 1:** Summary of reported outcomes in the study. ICER—incremental cost-effectiveness ratio; MoHP—Ministry of Health and Population.

Outcome	Description
Total costs	Change in total direct and indirect costs before vs. after the MoHP initiative
Direct costs	Change in medical costs before vs. after the MoHP initiative
Total indirect costs	Change in productivity losses for patients and caregivers before vs. after the MoHP initiative
Indirect patients’ costs	Change in productivity losses for patients before vs. after the MoHP initiative
Indirect caregivers’ costs	Change in productivity losses for caregivers before vs. after the MoHP initiative
QALYs gained/lost	Change in health outcomes associated with early vs. delayed intervention
ICER (societal perspective)	(Δ Direct costs + Δ Indirect patients’ costs + Δ Indirect caregivers’ costs)/Δ QALYs
ICER (payer perspective)	Δ Direct costs/Δ QALYs
Benefit–cost ratio (societal perspective)	Benefit–cost ratio = (ΔQALYs × GDP per capita)/(Δ Direct cost + Δ Indirect patients’ costs + Δ Indirect caregivers’ costs)

**Figure A2:**
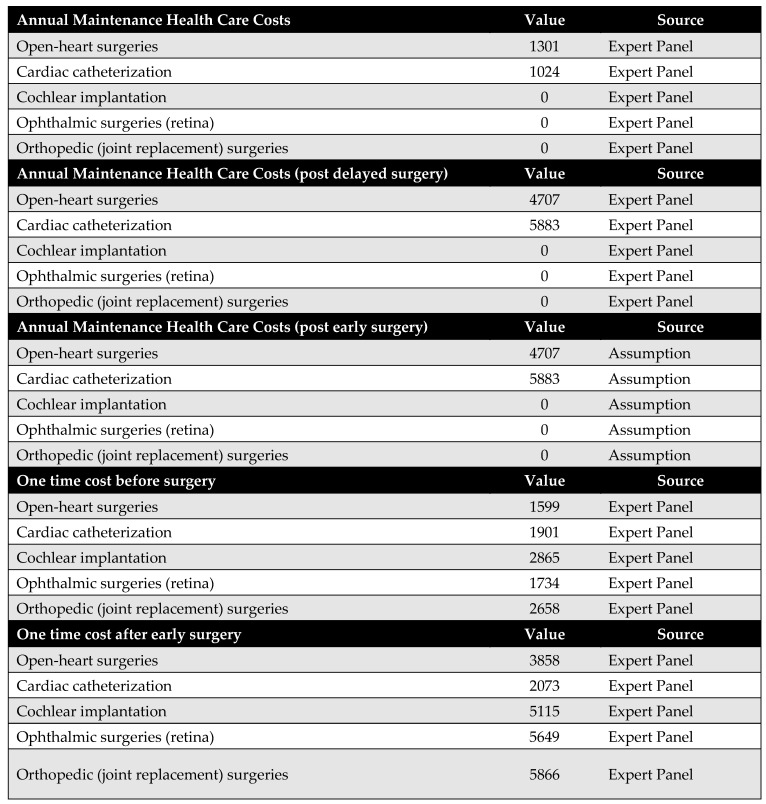
Medical costs. Annual maintenance healthcare costs post-early surgery were assumed to be equal to annual maintenance healthcare costs post-delayed surgery due to a lack of available data.

**Figure A3:**
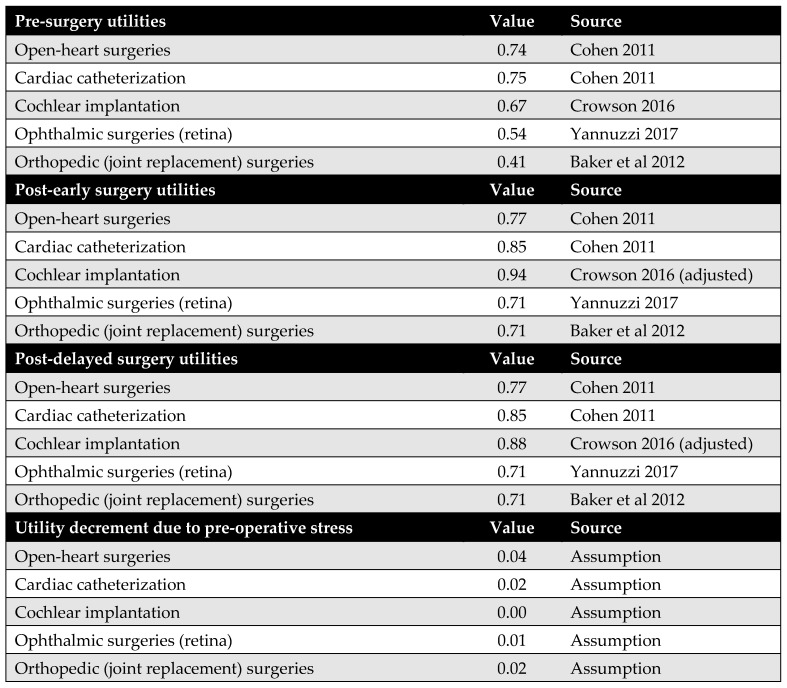
Model utilities and utility decrements. There was no evidence suggesting a difference in utility values between post-delayed and post-early surgery; therefore, these utilities were assumed to be equal. Additionally, due to the lack of literature on utility decrements associated with psychological stress from prolonged waiting times, these values were also assumed rather than empirically derived [23,32,33,34].

**Figure A4:**
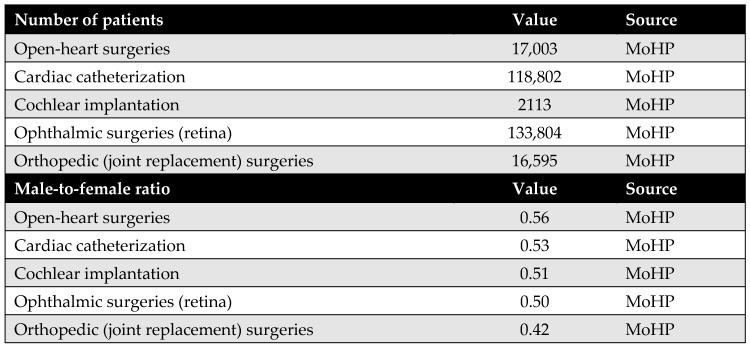
Number of patients who benefited from the MoHP initiative, and the male-to-female ratio.

**Figure A5:**
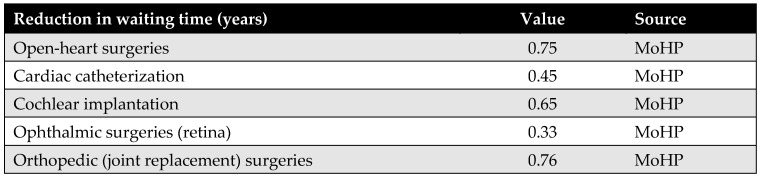
Reduction in waiting time in years.

**Figure A6:**
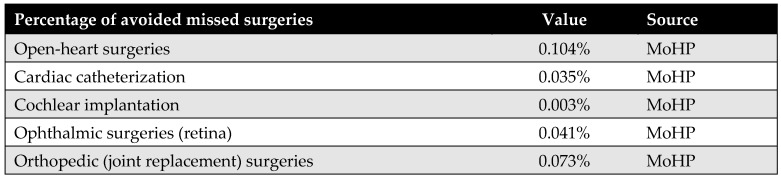
The percentage of avoided missed surgeries.

**Figure A7:**
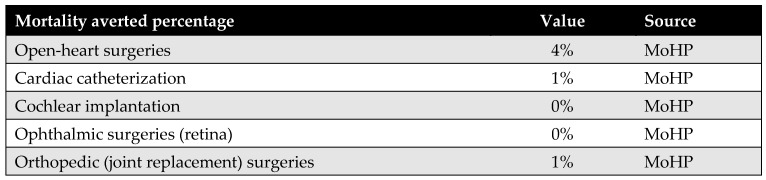
Percentage of mortality averted.

**Figure A8:**
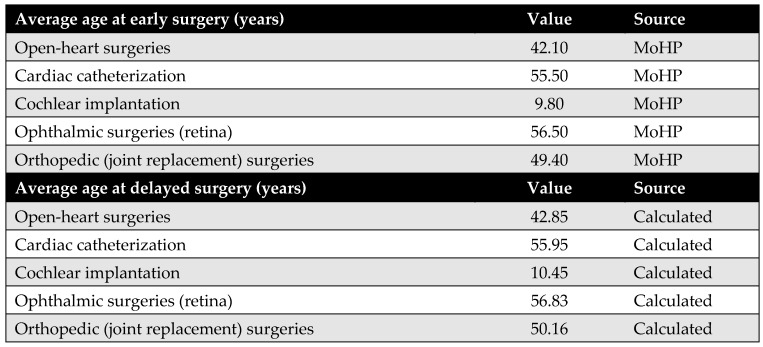
The average age at surgery.

**Figure A9:**
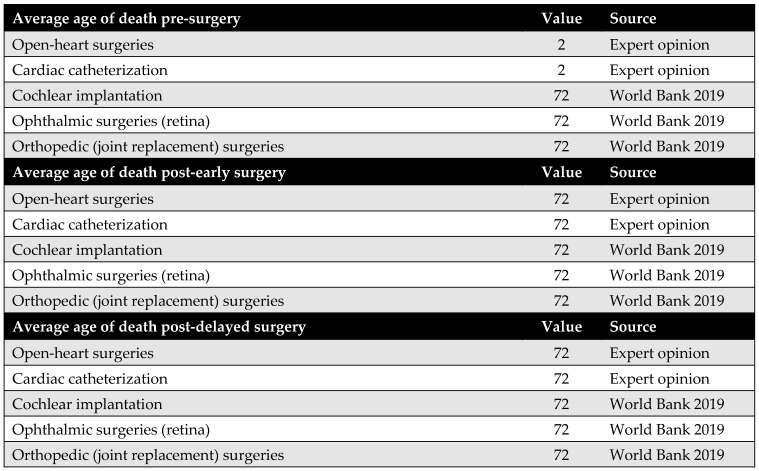
The average age at death [35].

**Figure A10:**
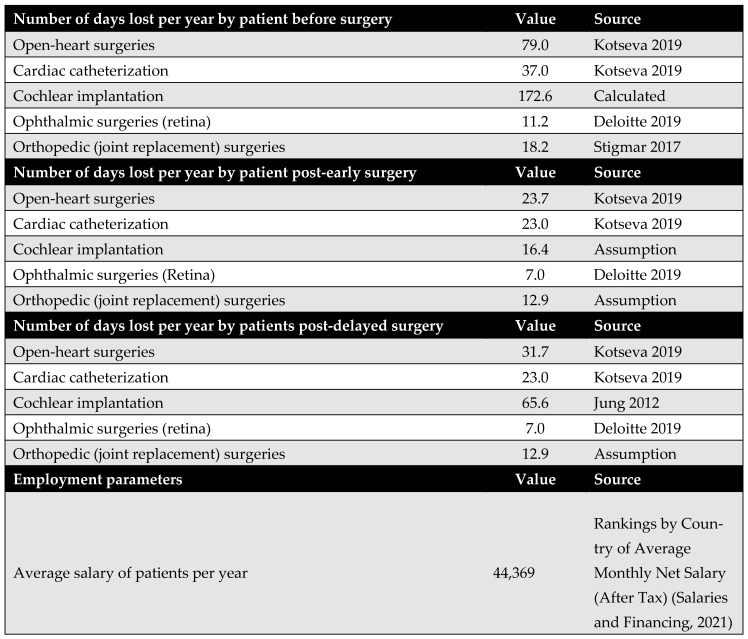
Days lost by the patients. Due to data limitations, productivity loss associated with cochlear implantation was estimated using proxy data on productivity loss related to hearing disabilities. Based on disability and unemployment statistics from the United Nations (2021), which indicate an 80% unemployment rate among individuals with disabilities, this rate was multiplied by the standard number of annual working days (216 days) to obtain the number of workdays lost. Based on these figures, assumptions were made regarding the number of days lost following early and delayed surgery, as well as the productivity loss incurred by caregivers [11,37,38,39,40].

**Figure A11:**
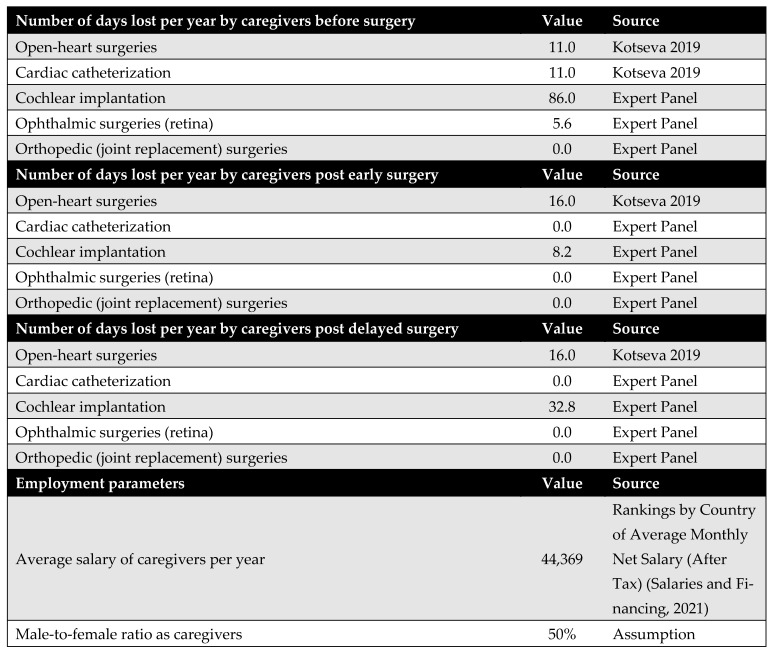
Days lost by the caregiver [11,40].

**Figure A12:**
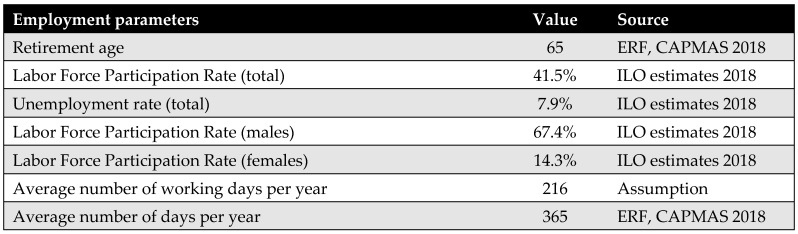
General productivity parameters [41,42].

**Figure A13:**
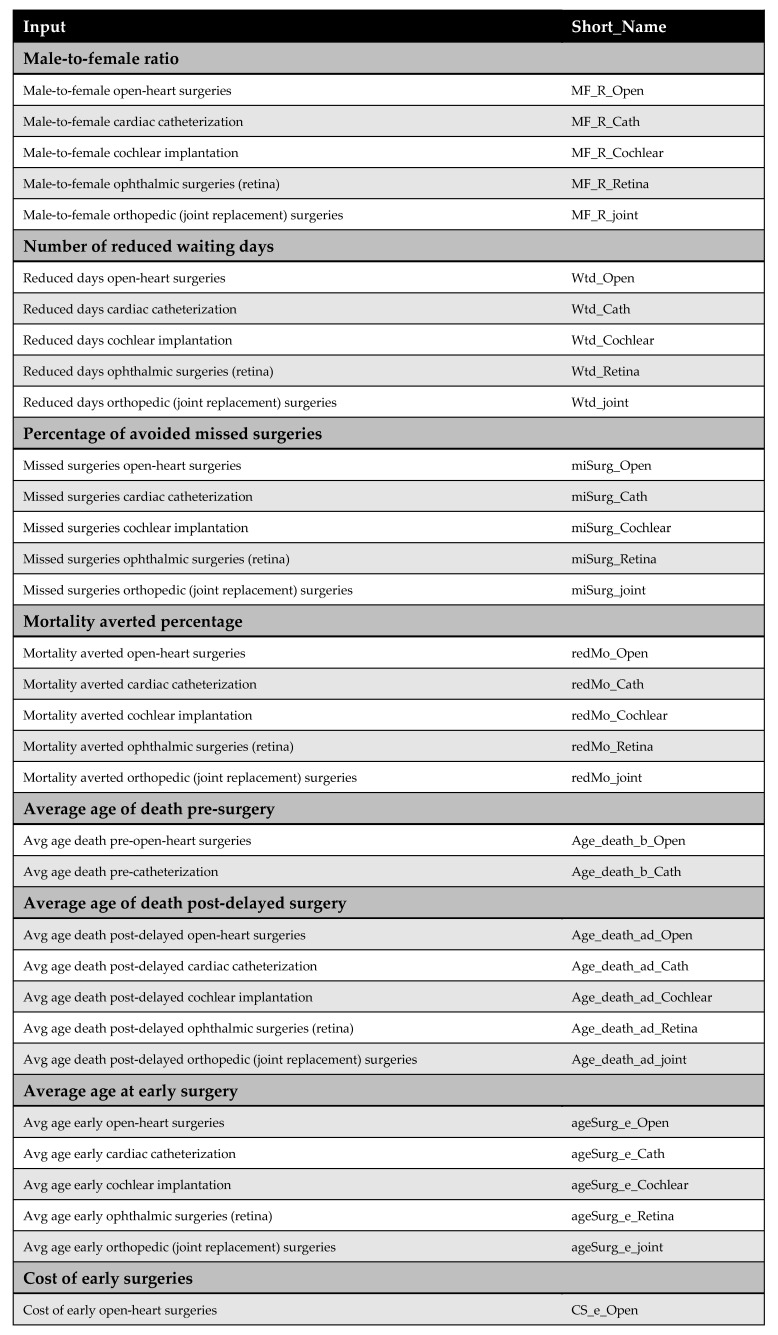

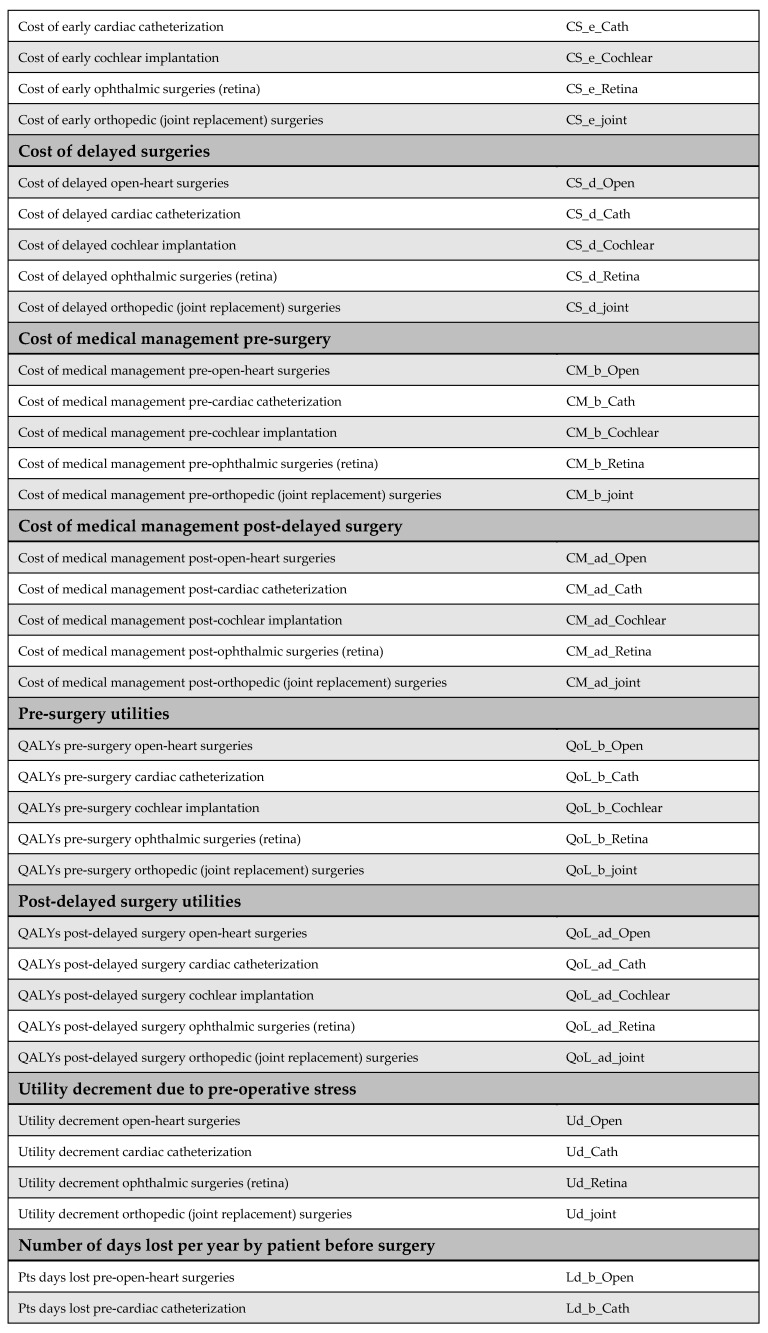

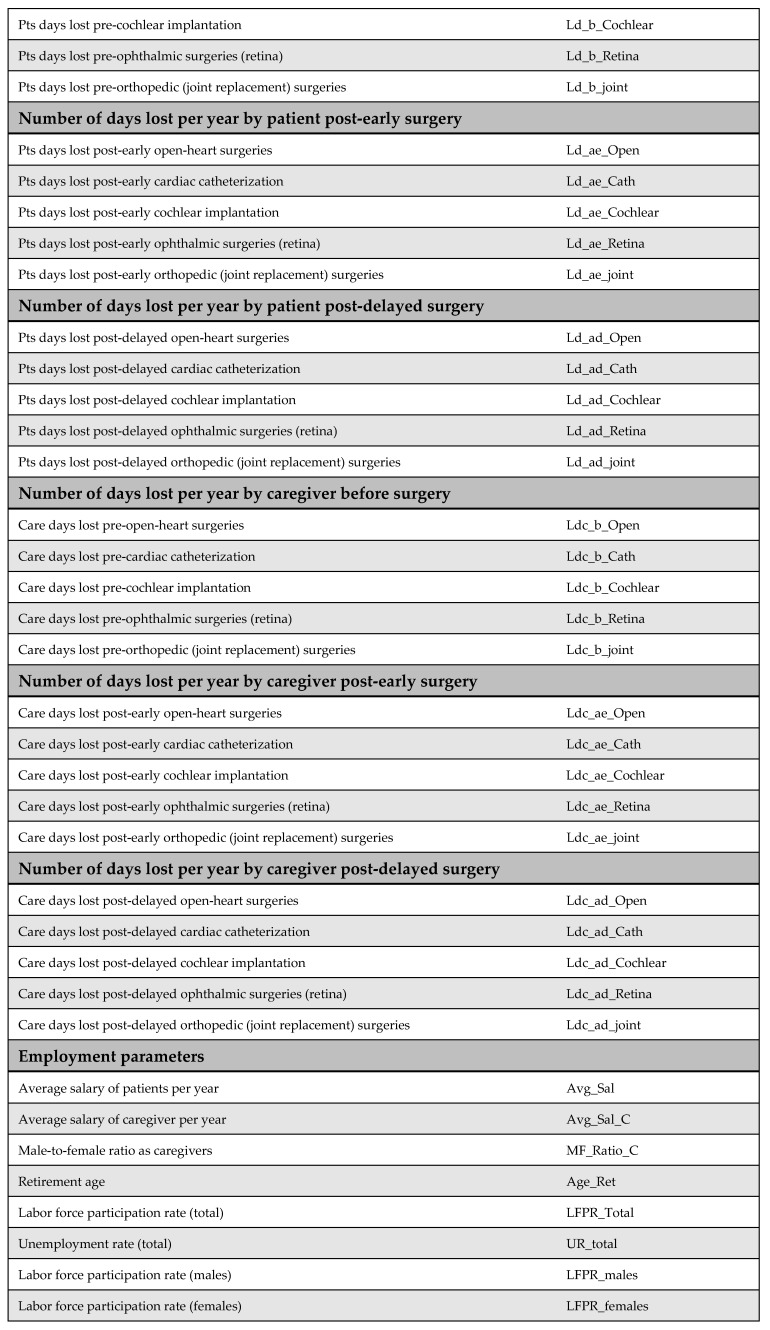

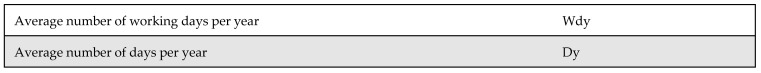
Model inputs included in the deterministic sensitivity analysis (DSA).

**Figure A14:**
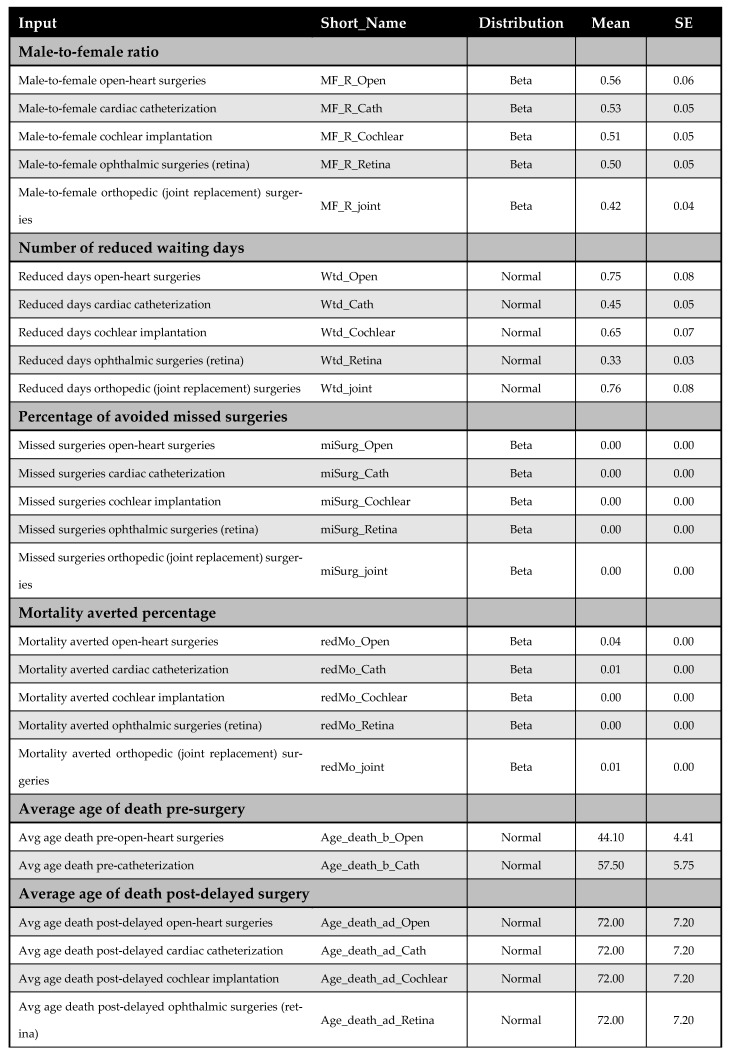

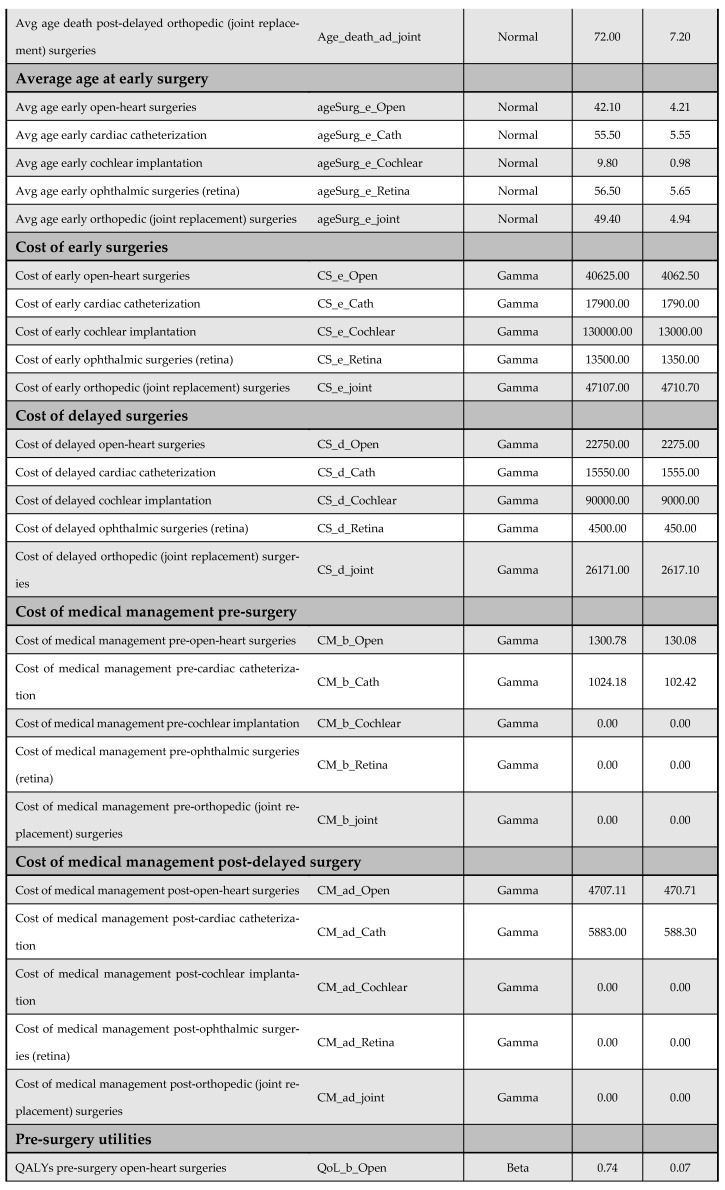

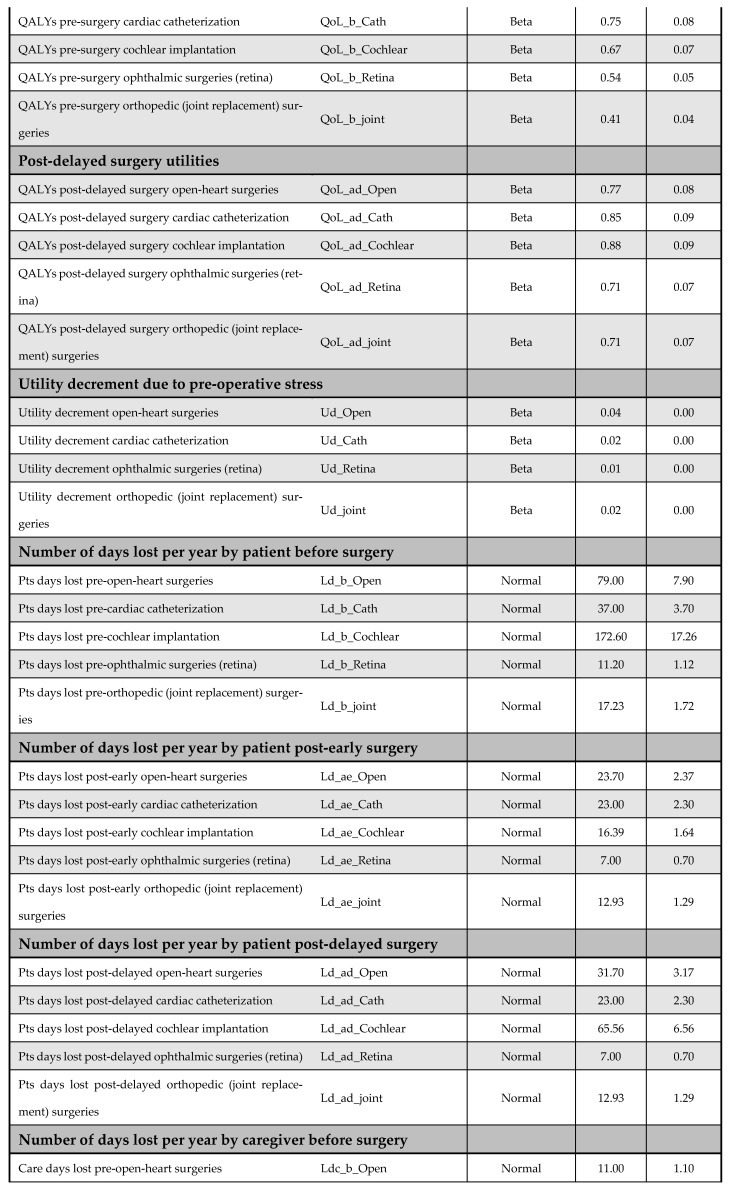

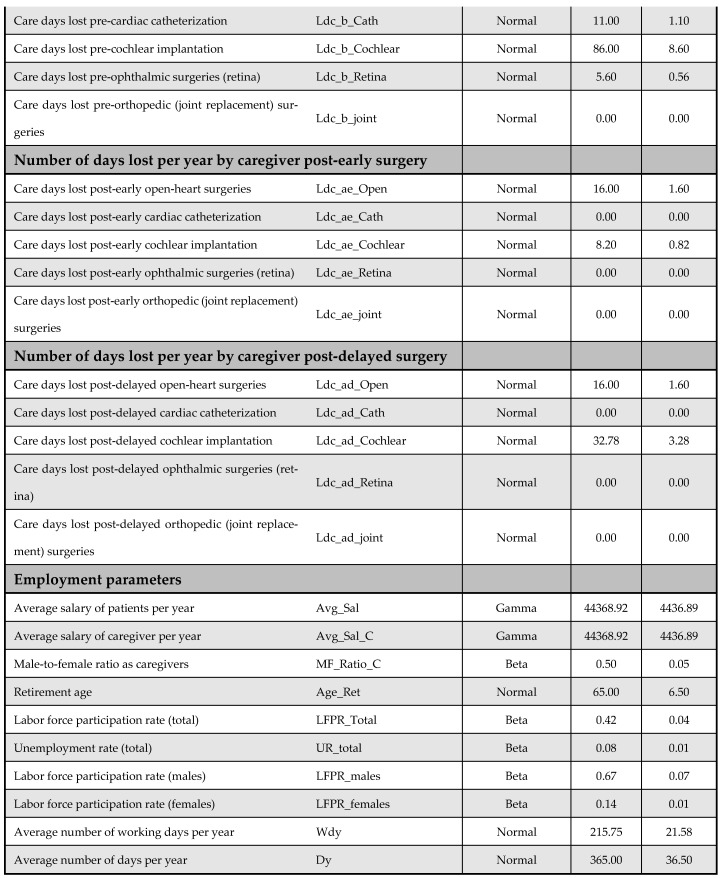
Model inputs included in the probabilistic sensitivity analysis (PSA).

**Figure A15:**
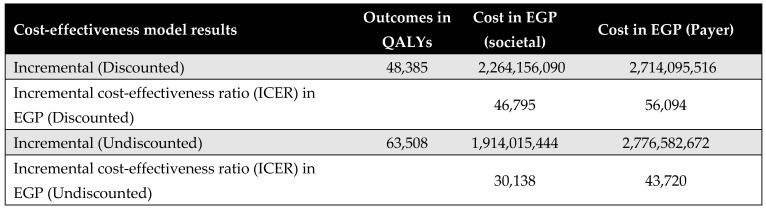
Aggregated results across all five priority interventions.

**Figure A16:**
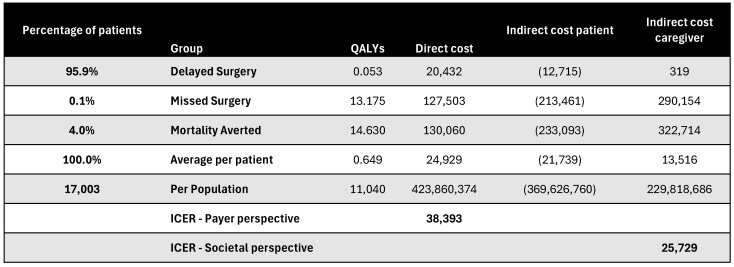
Discounted results for open-heart surgery. ICER—incremental cost-effectiveness ratio; QALY—quality-adjusted life year.

**Figure A17:**
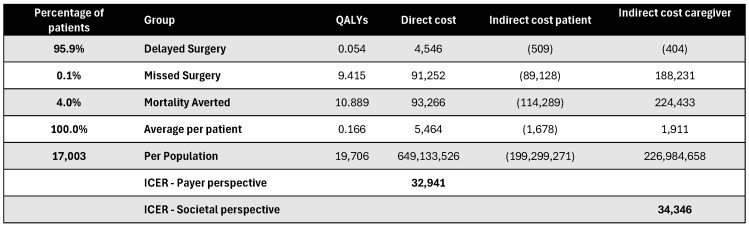
Discounted results for cardiac catheterization. ICER—incremental cost-effectiveness ratio; QALY—quality-adjusted life year.

**Figure A18:**
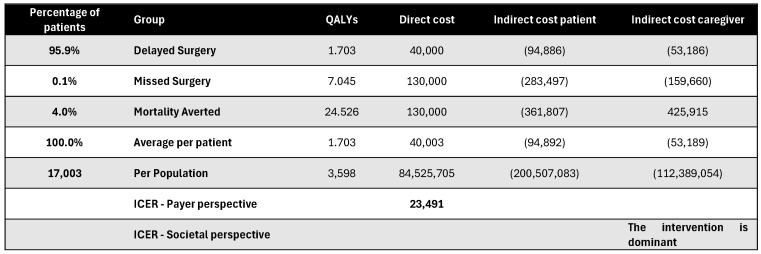
Discounted results for cochlear implantation. ICER—incremental cost-effectiveness ratio; QALY—quality-adjusted life year.

**Figure A19:**
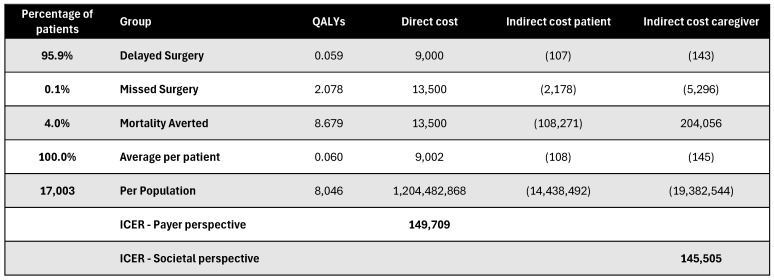
Discounted results for retina surgery. ICER—incremental cost-effectiveness ratio; QALY—quality-adjusted life year.

**Figure A20:**
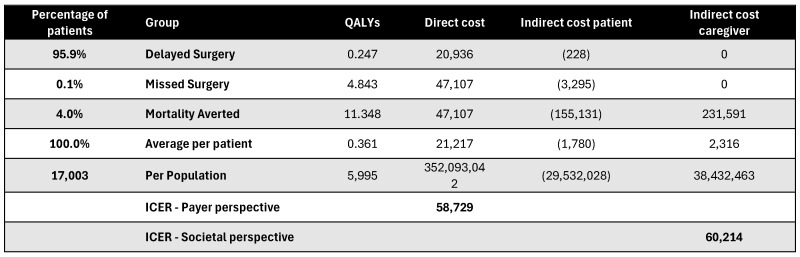
Discounted results for joint replacement surgery. ICER—incremental cost-effectiveness ratio; QALY—quality-adjusted life year.

**Figure A21:**
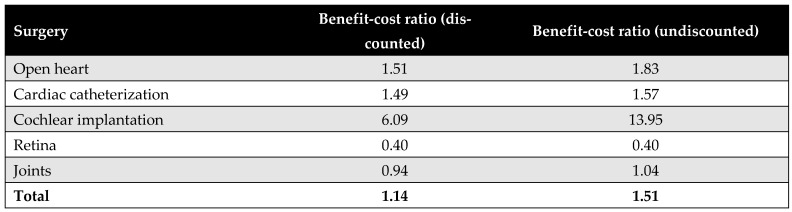
Benefit–cost ratios for each of the five priority interventions.

**Figure A22:**
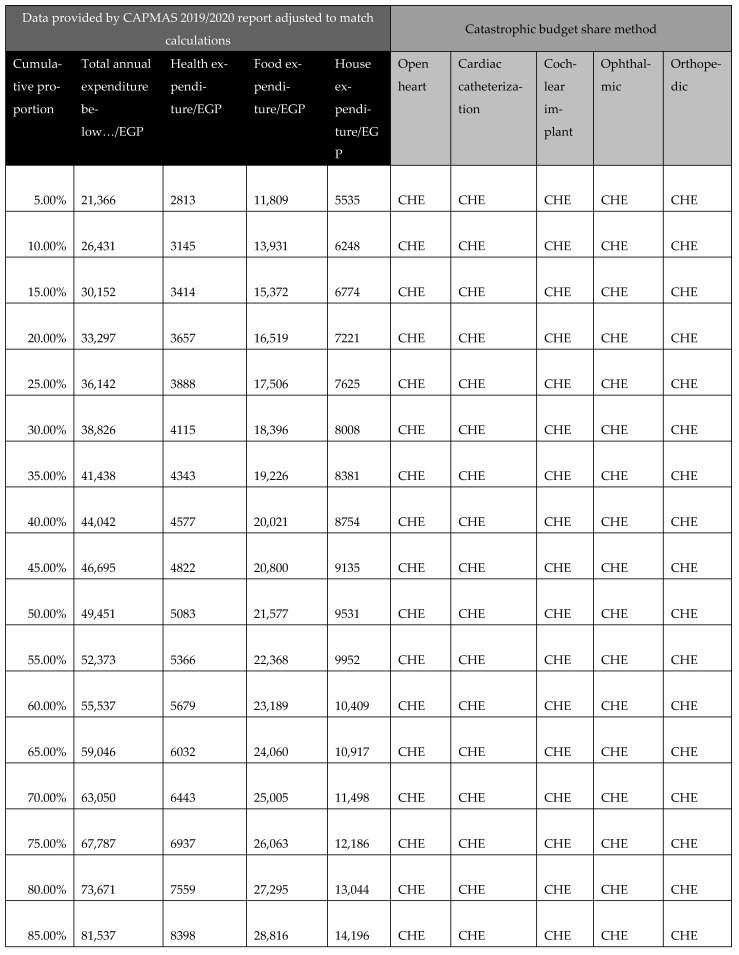

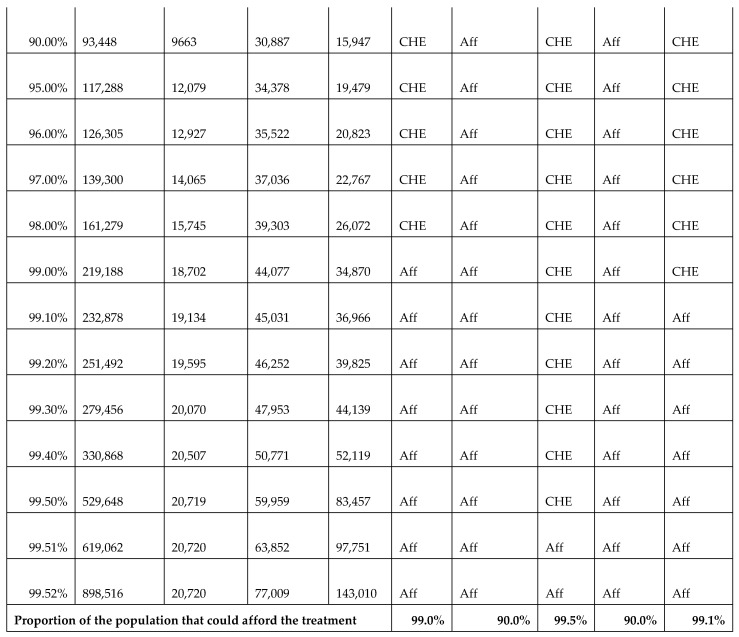
Calculations of catastrophic health expenditure (CHE) illustrating the affordability of surgeries across different income groups. Aff: affordable; CHE: catastrophic health expenditure.

**Figure A23:**
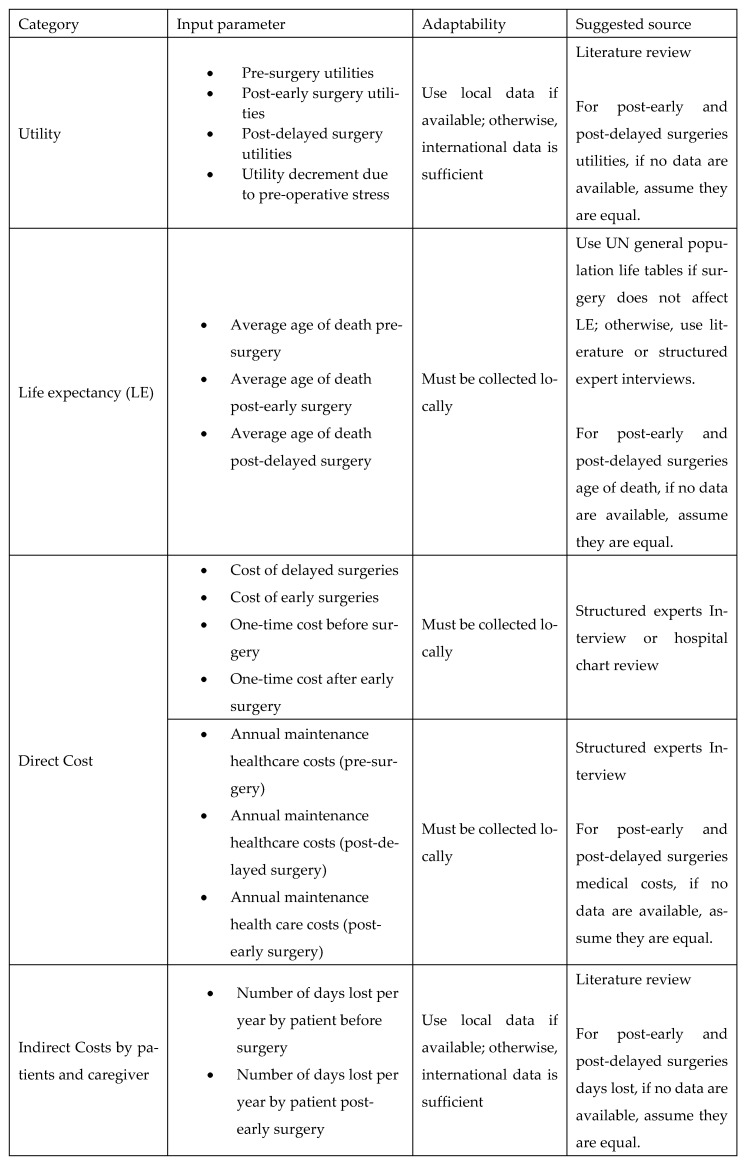

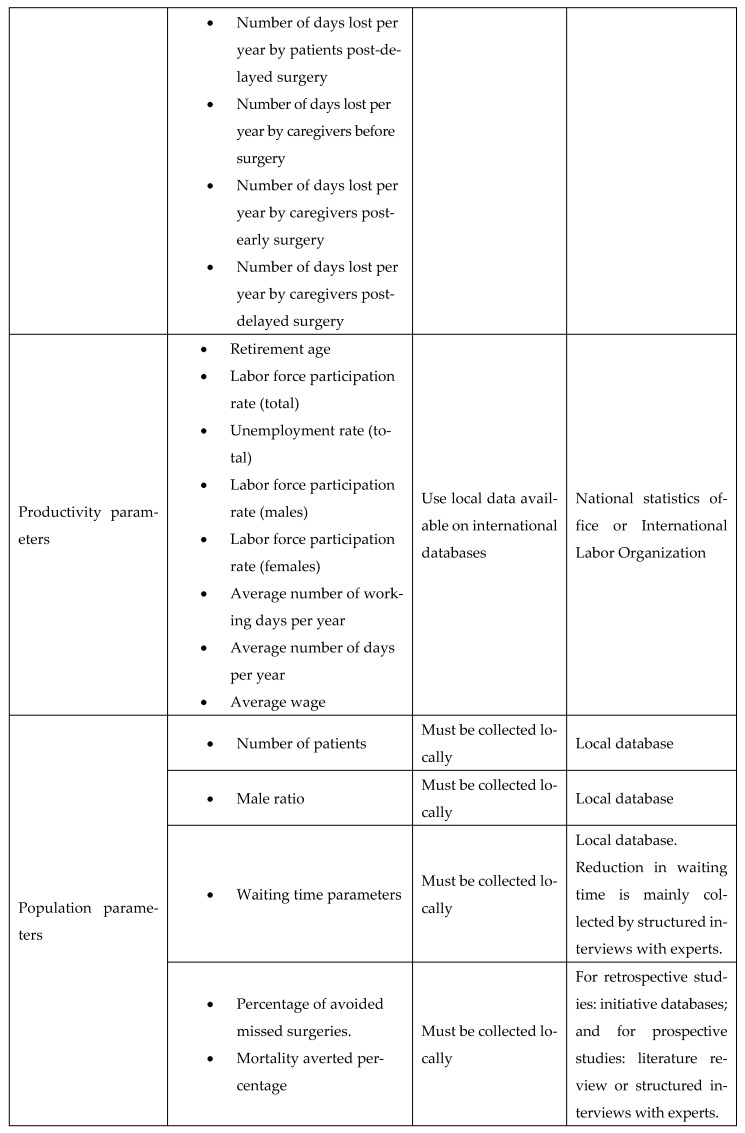
List of input parameters and suggested sources.

## Data Availability

The data presented in this study are available upon request from the corresponding author.

## Supplementary Materials

The following supporting information can be downloaded at: https://www.mdpi.com/article/10.3390/healthcare13131619/s1. M.1: Model of “The impact of the Egyptian MoHP initiative to eliminate the waiting lists” in Excel format.

## Abbreviations

The following abbreviations are used in this manuscript:

AMS	Avoided Missed Surgery
CAPMAS	Central Agency for Public Mobilization and Statistics
CHE	Catastrophic Health Expenditure
DCEA	Distributional Cost-Effectiveness Analysis
DSA	Deterministic Sensitivity Analysis
EGP	Egyptian Pound
GDP	Gross Domestic Product
ICER	Incremental Cost-Effectiveness Ratio
IRQG	Incremental Relative QALY Gain
LMIC	Low- and Middle-Income Countries
MA	Mortality Averted
MoHP	Ministry of Health and Population
OECD	Organization for Economic Cooperation and Development
OOP	Out-of-Pocket
PSA	Probabilistic Sensitivity Analysis
QALY	Quality-Adjusted Life Year
QoL	Quality of Life
RWT	Reduced Waiting Time
SE	Standard Error
SDG	Sustainable Development Goals
TAVI	Transcatheter Aortic Valve Implantation
UHC	Universal Health Coverage
WTP	Willingness-to-Pay

## Appendix A. Calculation of Direct Costs, Indirect Costs, and QALYs

This appendix summarizes the calculation of direct costs, indirect costs, and QALYs.

### Appendix A.1. Direct Costs

The total direct cost is calculated as follows:

$$\mathrm{Total}\;\mathrm{Direct}\;\mathrm{Cost}=C_{RW}+C_{AMS}+C_{MA}$$

where

$C_{RW}:Direct\;cost\;for\;Reduced\;Waiting\;time\;group;$
$C_{AMS}:Direct\;cost\;for\;Avoided\;Missed\;Surgery\;group;$
$C_{MA}:Direct\;cost\;for\;Mortality\;Averted\;group$.

### Appendix A.2. Direct Cost Breakdown

For each group, the direct cost is calculated as follows:

$$C_{RW}=\sum_{i=1}^{N}\left(\left(A_{ds}-A_{es}\right)\cdot \left(MC_{es}-MC_{bs}\right)+\left(A_{dd}-A_{ds}\right)\cdot \left(MC_{es}-MC_{ds}\right)\;+\left(A_{ed}-A_{dd}\right)\cdot MC_{es}+\left(C_{es}-C_{ds}\right)\right)$$

$$C_{AMS}=\sum_{i=1}^{N}\left(\left(A_{dbs}-A_{es}\right)\cdot \left(MC_{es}-MC_{bs}\right)+\left(A_{ed}-A_{dbs}\right)\cdot MC_{es}+C_{es}\right)$$

$$C_{MA}=\sum_{i=1}^{N}\left(\left(A_{dbs}-A_{es}\right)\cdot \left(MC_{es}-MC_{bs}\right)+\left(A_{ed}-A_{dbs}\right)\cdot MC_{es}+C_{es}\right)$$

where

N : Number of patients;
$A_{es}:Age\;at\;early\;surgery;$
$A_{ds}:Age\;at\;delayed\;surgery;$
$A_{dd}:Age\;at\;death\;after\;delayed\;surgery;$
$A_{ed}:Age\;at\;death\;after\;early\;surgery;$
$A_{dbs}:Age\;at\;death\;before\;surgery;$
$MC_{bs}:Medical\;costs\;before\;surgery;$
$MC_{es}:Medical\;costs\;after\;early\;surgery;$
$MC_{ds}:Medical\;costs\;after\;delayed\;surgery;$
$C_{es}:Cost\;of\;early\;surgery;$
$C_{ds}:Cost\;of\;delayed\;surgery$.

### Appendix A.3. Indirect Costs

The total indirect cost is calculated as follows:

$$Total\;Indirect\;Cost=IC_{P}+IC_{C}$$

where

$IC_{P}:Indirect\;cost\;by\;patients;$
$IC_{C}:Indirect\;cost\;by\;caregivers$.

### Appendix A.4. Indirect Cost Breakdown

For each group, the indirect cost is calculated as follows:

$$IC_{P}=\sum_{i=1}^{N}\left(\frac{\left(D_{aes}-D_{bs}\right)}{WD}\cdot \left(A_{ds}-A_{es}\right)+\frac{\left(D_{aes}-D_{ds}\right)}{WD}\cdot \left(A_{dd}-A_{ds}\right)\;+\left(\frac{D_{aes}}{WD}-1\right)\cdot \left(A_{ed}-A_{dd}\right)\right)\cdot E_{P}$$

$$IC_{AMS}=\sum_{i=1}^{N}\left(\frac{\left(D_{aes}-D_{bs}\right)}{WD}\cdot \left(A_{dbs}-A_{es}\right)+\left(\frac{D_{aes}}{WD}-1\right)\cdot \left(A_{ed}-A_{dbs}\right)\right)\cdot E_{P}$$

$$IC_{MA}=\sum_{i=1}^{N}\left(\frac{\left(D_{aes}-D_{bs}\right)}{WD}\cdot \left(A_{dbs}-A_{es}\right)+\left(\frac{D_{aes}}{WD}-1\right)\cdot \left(A_{ed}-A_{dbs}\right)\right)\cdot E_{P}$$

where

$D_{aes}:Days\;lost\;after\;early\;surgery;$
$D_{bs}:Days\;lost\;before\;surgery;$
$D_{ds}:Days\;lost\;after\;delayed\;surgery;$
WD : Number of working days per year;
$E_{P}:Employment\;parameters\;\left(adjusted\;by\;gender,\;labor\;force\;participation\;rate,\;and\;unemployment\;rate\right)$.
For caregivers the corresponding expressions are as follows:

$$IC_{C}=\sum_{i=1}^{N}\left(\frac{\left(D_{ces}-D_{cbs}\right)}{WD}\cdot \left(A_{ds}-A_{es}\right)+\frac{\left(D_{ces}-D_{cds}\right)}{WD}\cdot \left(A_{dd}-A_{ds}\right)+1\;-\left(\frac{D_{ces}}{WD}\right)\cdot \left(A_{ed}-A_{dd}\right)\right)\cdot E_{C}$$

$$IC_{AMS}=\sum_{i=1}^{N}\left(\frac{\left(D_{ces}-D_{cbs}\right)}{WD}\cdot \left(A_{dbs}-A_{es}\right)+1-\left(\frac{D_{ces}}{WD}\right)\cdot \left(A_{ed}-A_{dbs}\right)\right)\cdot E_{C}$$

$$IC_{MA}=\sum_{i=1}^{N}\left(\frac{\left(D_{ces}-D_{cbs}\right)}{WD}\cdot \left(A_{dbs}-A_{es}\right)+1-\left(\frac{D_{ces}}{WD}\right)\cdot \left(A_{ed}-A_{dbs}\right)\right)\cdot E_{C}$$

where

$D_{ces}:Days\;lost\;by\;caregivers\;after\;early\;surgery;$
$D_{cbs}:Days\;lost\;by\;caregivers\;before\;surgery;$
$D_{cds}:Days\;lost\;by\;care\;givers\;after\;delayed\;surgery;$
$E_{C}:Employment\;parameters\;for\;caregivers$.

### Appendix A.5. Quality-Adjusted Life Years (QALYs)

The total QALYs gained are calculated as follows:

$$\mathrm{Total}\;\mathrm{QALYs}=Q_{RW}+Q_{AMS}+Q_{MA}$$

where

$Q_{RW}:QALYs\;gained\;for\;Reduced\;Waiting\;time\;group;$
$Q_{AMS}:QALYs\;gained\;for\;Avoided\;Missed\;Surgery\;group;$
$Q_{MA}:QALYs\;gained\;for\;Mortality\;Averted\;group$.

### Appendix A.6. QALYs Breakdown

For each group, the QALYs are calculated as follows:

$$Q_{RW}=\sum_{i=1}^{N}\left(\left(Q_{aes}-Q_{bs}\right)\cdot \left(A_{ds}-A_{es}\right)+\left(Q_{aes}-Q_{ds}\right)\cdot \left(A_{dd}-A_{ds}\right)+Q_{aes}\cdot \left(A_{ed}-A_{dd}\right)\right)\cdot Ud$$

$$Q_{AMS}=\sum_{i=1}^{N}\left(\left(Q_{aes}-Q_{bs}\right)\cdot \left(A_{dbs}-A_{es}\right)+Q_{aes}\cdot \left(A_{ed}-A_{dbs}\right)\right)$$

$$Q_{MA}=\sum_{i=1}^{N}\left(\left(Q_{aes}-Q_{bs}\right)\cdot \left(A_{dbs}-A_{es}\right)+Q_{aes}\cdot \left(A_{ed}-A_{dbs}\right)\right)$$

where

$Q_{aes}:QALYs\;after\;early\;surgery;$
$Q_{bs}:QALYs\;before\;surgery;$
$Q_{ds}:QALYs\;after\;delayed\;surgery$;
Ud:Utility decrement due to pre−operative stress.

## Appendix B. Cost Parameters

### Appendix B.1. Surgical Costs

Surgical costs were calculated based on comprehensive case-based payments, which include the surgeon’s fee, medical equipment, prostheses (if applicable), immediate pre- and post-operative investigations, and hospitalization during the perioperative period. The costs of surgeries differed before and after the MoHP initiative (Figure A1). All pricing data were obtained from the MoHP and are reported in 2021 EGP.

### Appendix B.2. Medical Costs

Medical costs represent a key parameter in the model and are critical for accurately assessing the cost-effectiveness of the MoHP initiative. To capture variations in treatment expenses, costs were estimated using a structured data collection tool and input from an expert panel representing each surgical specialty. For each procedure, one to two clinical experts were interviewed independently to complete the structured tool and respond to open-ended questions, ensuring accurate reporting of resource and medication utilization.

The MoHP provided official price lists for the relevant services, which were updated to reflect 2021 EGP values. These prices were used to calculate both annual maintenance costs (pre- and post-procedure) and one-time medical costs not included in the surgical package. For medications, current market prices were used to ensure accurate cost estimation. One-time costs are defined as those medical expenses incurred before or after the surgery that are not included in the bundled surgical payment. In the original model design, post-operative annual maintenance costs following early surgery were assumed to differ from preoperative maintenance costs. However, due to insufficient data, we assumed these costs to be equal in this version of the model.

## Appendix C. Utility Parameters

Health outcomes in this study are measured using QALYs. QALYs were calculated by multiplying the health utility associated with each health state by the duration spent in that state. For example, the QALYs accumulated before surgery were determined by multiplying the time spent awaiting surgery by the utility value of the pre-surgery health state.

Health utility values for both pre-surgery and post-surgery states were sourced from the published literature. The original model design accounted for the possibility that post-delayed surgery utilities might differ from those of post-early surgery. However, due to the lack of empirical evidence supporting this distinction, we assumed that both post-surgery utilities were equal in this version of the model.

Additionally, no studies were identified that quantify utility decrements resulting from psychological stress associated with long surgical wait times. As such, this potential impact could not be incorporated into the model.

## Appendix D. Population Parameters

### Appendix D.1. Number of Patients

The number of patients was obtained from the MoHP database, representing the actual number of individuals who underwent the selected procedures as part of the MoHP initiative between January 2019 and February 2020. In addition, male-to-female ratios for each procedure were also sourced from the MoHP to complement the patient data.

### Appendix D.2. Waiting Time

Waiting time is a key parameter in the model, defined as the difference in average waiting time before and after the MoHP initiative, measured in years. This data was obtained from the MoHP.

### Appendix D.3. Percentage of Avoided Missed Surgeries

This parameter represents the proportion of patients who, in the absence of the MoHP initiative, would likely have missed their necessary surgical procedures due to excessive waiting times. The MoHP initiative effectively enabled these patients to receive timely care, transitioning them from the missed surgery category to the group of patients who successfully received treatment. This percentage was calculated using data from the MoHP databases.

### Appendix D.4. Percentage of Mortality Averted

The percentage of mortality averted reflects the proportion of patients who received surgical procedures through the MoHP initiative but who would have died while waiting without timely intervention. By facilitating earlier access to care, the MoHP initiative effectively saved these lives, transitioning patients from the mortality category to the group successfully treated. This parameter was calculated using data from the MoHP databases.

### Appendix D.5. The Average Age at Surgery

The average age of patients undergoing each procedure—both before and after the MoHP initiative—is a crucial parameter for calculating differences in costs, health outcomes, and the timeframe of the model. The average age at the time of early surgery (during the MoHP initiative) was obtained from the MoHP databases. In contrast, the average age at delayed surgery (prior to the MoHP initiative) was estimated by adding the average waiting time (in years) to the average age at early surgery.

### Appendix D.6. The Average Age at Death

The average age at death, together with the average age at surgery, is used to estimate patients’ lifetime before surgery, after delayed surgery (pre-MoHP initiative), and after early surgery (post-MoHP initiative). Based on expert panel input, it was assumed that cochlear implantation, ophthalmic surgeries, and joint replacement do not influence life expectancy. In these cases, the average age at death—whether surgery was delayed or performed early—is assumed to align with the general population’s life expectancy in Egypt. In contrast, open-heart surgery and cardiac catheterization were considered to impact life expectancy, particularly when performed in a timely manner. Therefore, the average age at death differs depending on whether these procedures were performed early or delayed.

## Appendix E. Productivity Parameters

### Appendix E.1. Patients’ Productivity Parameters

Productivity loss among patients is a major cost driver in the model from the societal perspective. The number of workdays lost before and after surgery was obtained from the literature for all procedures, apart from cochlear implantation. Despite being a life-changing intervention for children and their families, there is limited research quantifying the impact of cochlear implantation on productivity loss in terms of workdays.

To address this gap, the productivity loss for cochlear implantation was estimated based on the impact of hearing disability on employment. According to Disability and Employment | United Nations Enable (2021) [36], the unemployment rate among people with disabilities is approximately 80%. This rate was applied to the standard number of annual working days (216 days) to estimate the number of workdays lost. Using these figures, assumptions were made regarding days lost before and after both early and delayed surgery, as well as productivity loss for caregivers. Although the model was originally designed to distinguish between productivity losses following early versus delayed surgery, identical figures were used in this version due to the lack of specific supporting data in the literature.

### Appendix E.2. Caregivers’ Productivity Parameters

Productivity loss among caregivers is another significant cost driver from the societal perspective. However, the available literature on this topic is limited. Empirical data were found only for open-heart surgery and cardiac catheterization. For the remaining procedures, including cochlear implantation, ophthalmic surgery, and joint replacement, productivity losses were estimated based on expert opinion due to the lack of published data.

## Appendix F. DSA and PSA Inputs

Figure A13 and Figure A14 outline model inputs included in the DSA and PSA, respectively.

## Appendix G. Individual Results for Each Intervention

While Figure A15 shows aggregated results across all five priority interventions, Figure A16, Figure A17, Figure A18, Figure A19 and Figure A20 present the results for each of the five priority interventions individually. Reducing waiting times for open-heart surgery and cardiac catheterization was found to be cost-effective from both the societal and payer perspectives (Figure A16 and Figure A17). Cochlear implantation was dominant—i.e., more effective and less costly—from the societal perspective, and cost-effective from the payer perspective (Figure A18). In contrast, reducing waiting times for ophthalmic (retinal) surgery was not cost-effective from either perspective (Figure A19). Joint replacement surgery was found to be borderline cost-effective from both the societal and payer perspectives, with ICERs close to the established threshold (Figure A20).

Figure A21 presents the benefit–cost ratios for each of the five priority interventions. On average, the MoHP initiative delivered a return of 1.14 EGP per EGP spent in the discounted scenario, and EGP 1.51 in the undiscounted scenario. Among the procedures, cochlear implantation demonstrated the highest return on investment, with each EGP invested yielding an estimated return of approximately EGP 6.

## Appendix H. Calculations of Catastrophic Health Expenditure (CHE)

In calculating CHE, we used data from the Survey of Income, Expenditure, and Consumption 2019/2020, published by CAPMAS (Central Agency for Public Mobilization and Statistics; available from https://censusinfo.capmas.gov.eg/Metadata-ar-v4.2/index.php/catalog/1773 accessed on 2 July 2025). The report details income and expenditure patterns across different population segments, indicating variations in income levels. Using the survey data, we determined the surgery affordability for different income groups, enabling us to estimate the proportion of the population capable of affording elective surgeries without financial hardship and the proportion likely to experience CHE. Further details of these calculations are presented in Figure A22.

## Appendix I. Adaptation Guide

A key goal of our study was to ensure simplicity and adaptability. To facilitate this, we developed an adaptation guide (Figure A23). The practical use of the adaptation guide was demonstrated by the scenario in Appendix J, making the cost-utility model widely applicable across LMIC settings.

## Appendix J. Example Scenario: Adaptation Guide for Elective Surgery Waiting List Reduction Initiative in a Low/Middle-Income Country (LMIC)

The LMIC faces substantial delays in elective surgical procedures, including orthopedic joint replacements and cardiac surgeries, resulting in adverse health outcomes and significant financial burdens for patients. To address this, the LMIC’s Ministry of Health (MoH) aims to implement a national initiative similar to Egypt’s surgical waiting time reduction program, i.e., the MoHP initiative. Given the limited local data availability, the adaptation guide provided (Figure A23, Appendix I) will be used to facilitate a robust economic evaluation of this initiative.

### Appendix J.1. Step-by-Step Application of the Adaptation Guide

#### Appendix J.1.1. Utility Parameters

Due to the limited local health-utility data, the LMIC’s researchers will perform a targeted literature review to source pre-surgery and post-surgery utility values, prioritizing studies conducted in similar LMIC settings. If such data are unavailable, global averages or assumptions (e.g., equal utility for post-early and post-delayed surgery states) may be used as per the adaptation guide.

#### Appendix J.1.2. Life Expectancy (LE)

To estimate life expectancy parameters, the researchers will refer to the United Nations general population life tables for the LMIC. For life-saving procedures (e.g., cardiac surgeries), local clinical experts will provide structured interviews to estimate impacts on life expectancy for early versus delayed interventions.

#### Appendix J.1.3. Direct Cost Parameters

Direct surgical costs, including pre- and post-operative management, will be obtained through structured interviews with clinical experts from representative hospitals in LMIC. If precise cost breakdowns are unavailable, average costs from major public hospitals in the capital of LMIC will serve as a proxy.

#### Appendix J.1.4. Indirect Costs (Productivity Losses)

For indirect productivity costs (days lost by patients and caregivers), the LMIC’s researchers will first search for local literature. In the absence of LMIC-specific productivity loss data, they will adopt estimates from similar regions, e.g., African/Asian LMICs, or international studies, as recommended by the adaptation guide. Productivity parameters, such as average wages and employment statistics, will be sourced from the LMIC’s national statistics bureau and the International Labour Organization.

#### Appendix J.1.5. Population Parameters

The LMIC’s MoH patient databases will provide patient numbers, gender ratios, waiting times, and intervention-specific data. For parameters not readily available (e.g., percentage of avoided missed surgeries, mortality averted), structured expert interviews will be conducted with local specialists who regularly manage waiting lists.

### Appendix J.2. Outcome

Using this structured adaptation approach, the LMIC will establish a reliable economic evaluation framework tailored to local conditions, thereby providing actionable insights for policymakers. The resulting evaluation will demonstrate the health and economic impacts of reducing elective surgical waiting times and support informed decisions on healthcare investments.
